# Association between physical activity level and diabetes incidence among Chinese middle-aged and older adults: a cross-sectional study from the China health and retirement longitudinal study

**DOI:** 10.3389/fpubh.2024.1430229

**Published:** 2024-08-09

**Authors:** Yunqing Zhang, Fanhao Meng, Xueyin Fei, Ke Wang, Yigao Wu, Xueting Wang

**Affiliations:** ^1^China Basketball College, Beijing Sport University, Beijing, China; ^2^School of Strength and Conditioning, Beijing Sport University, Beijing, China; ^3^Sport Science School, Beijing Sport University, Beijing, China; ^4^China Institute of Sports and Health Science, Beijing Sport University, Beijing, China; ^5^Department of Physical Education, Jiangsu Normal University, Xuzhou, China

**Keywords:** physical activity, diabetes, middle-aged and older adults, China, CHARLS

## Abstract

**Background:**

It has been shown that diabetes is associated with insufficient physical activity among middle-aged and older adults, but the association between different physical activity levels (PAL) and diabetes incidence needs to be further explored.

**Objective:**

This study aims to explore the correlation and dose–response relationship between different PAL and the diabetes incidence in middle-aged and older adults.

**Methods:**

Utilizing data from the 2018 China Health and Retirement Longitudinal Study (CHARLS), this cross-sectional analysis included 17,226 middle-aged and older adults aged 45 and above. Binary logistic regression models and restricted cubic spline (RCS) were used to explore the correlation and dose–response relationship between different PAL and the incidence of diabetes in the total middle-aged and older adults population as well as in subgroups. Sensitivity analyses were also performed to verify the robustness of the findings.

**Results:**

In the entire study population, compared with the lowest PAL, participants in the third and fourth quartiles PAL saw diabetes incidence significantly reduced by 16% (*p* = 0.005) and 33% (*p* < 0.001), respectively (*p*_for trend_ < 0.001). In subgroup analyses, the fourth quartile PAL significantly reduced the diabetes incidence among females, individuals aged 60–69, and rural residents by 25% (*p* = 0.011), 38% (*p* < 0.001) and 28% (*p* < 0.001), respectively. For males, middle-aged (45–59 years), and urban residents, the third quartile PAL reduced diabetes incidence by 22% (*p* = 0.004), 24% (*p* = 0.012), 21% (*p* = 0.013), respectively. When the fourth quartile PAL was reached, the diabetes incidence was significantly reduced in these populations by 41% (*p* < 0.001), 39% (*p* < 0.001), and 41% (*p* < 0.001), respectively. There was a negative dose–response relationship between physical activity and diabetes incidence in specific Chinese middle-aged and older adults population. In addition, sensitivity analyses indicated the robustness of the findings.

**Conclusion:**

Higher PAL was associated with lower diabetes incidence in specific Chinese middle-aged and older adults population. It is feasible to use physical activity to predict diabetes incidence in this demographic, and high PAL may be an effective means of preventing and controlling diabetes.

## 1 Introduction

Diabetes is one of the leading causes of death worldwide, with an estimated 4.2 million deaths in 2019 (1). By 2030, global diabetes diagnoses are projected to reach 578 million, surging to an anticipated 700 million by 2045 (2). Diabetes has a high prevalence in the middle-aged and older adults population (3, 4) and exert a huge impact on the health of them; it is a contributing factor to a number of diseases such as myocardial infarction, dementia, and depression (5–7), and can lead to a variety of complications such as fractures, falls, and pain (8–11), which not only leads to their moodiness and irritability, and can even lead to frailty, disability and suicide. Additionally, population aging is a problem faced by the whole world, especially China. Currently, population aging in China is intensifying, and it is expected that by 2050, China’s older adults population over 65 years old will reach 400 million, of which 150 million will be over 80 years old (12, 13). As one of the fastest aging countries in the world, the number of older adults people with diabetes will continue to grow in China. This will not only seriously affect people’s quality of life, but also bring a heavy economic burden to the affected families and society (14). Consequently, how to prevent and control diabetes in middle-aged and older adults people in a healthy and effective way has become a hot topic in the field of public health.

Research indicates that insufficient physical activity contributes to diabetes and its complications among middle-aged and older adults individuals, while regular physical activity can prevent diabetes in this demographic (15–17). Physical activity, defined as any movement that expends energy through musculoskeletal contraction and relaxation, encompasses all daily physical movements, and it is a broad concept that includes more specific parameters such as type, intensity, duration and frequency (18). While some research has identified a negative association between physical activity and diabetes incidence (19), other studies suggest that moderate physical activity level (PAL) reduces diabetes incidence more effectively than other levels (20). Currently, the association between the protective effect of physical activity on the incidence of diabetes in middle-aged and older adults people has been demonstrated. However, the association between different PAL and diabetes incidence remains unclear, especially given the limited studies within this demographic in China. Moreover, it has been shown that the prevalence of diabetes is largely influenced by age, gender, and residency (21), but it is also unclear whether there are age, gender, and residency differences in the association between physical activity and diabetes incidence.

Further research is needed to investigate the association between varying PAL and diabetes incidence in Chinese middle-aged and older adults populations. This study aimed to explore the correlation and dose–response relationship between various PAL and diabetes incidence, assessing differences by age, gender, and residency. Exploring this relationship may facilitate early prediction of diabetes and inform effective interventions. Therefore, this study investigated the association between different PAL and the diabetes incidence in the middle-aged and older adults population and the differences in gender, age, and residency, thus providing a scientific basis for the prediction and treatment of diabetes in this groups.

## 2 Methods

### 2.1 Data sources and participants

This is a cross-sectional study based on data from the 2018 China Health and Retirement Longitudinal Study (CHARLS). The survey is a nationally representative community-based demographic survey of middle-aged and older adults people in China, conducted by the China National Development Research Institute (CNDI) in collaboration with the China Social Science Research Centre (CSRC) of Peking University (22). Employing a stratified, multi-stage,probability sampling method proportional to population size, the survey sampled individuals aged 45 and above from 150 counties and 450 communities (villages) across 28 Chinese provinces (autonomous regions and municipalities). It collected demographic, economic, pension, and health data The CHARLS database has good reliability and validity, accurately representing China’s middle-aged and older adults populations, and it has been used by many scholars to conduct health-related research in recent years (23). Additionally, Ethical approval for CHARLS was granted by Peking University’s Biomedical Ethics Review Committee (Approval No.: IRB00001052-11015) (22). Since the data is publicly available, no further ethical approval was necessary for this study.

According to the definition of middle-aged and older adults in China, respondents aged 45 years and above were included as the research subjects. Eligible participants must have complete data on physical activity, diabetes status, and relevant covariates. Therefore, respondents under 45 or with missing data in these areas were excluded. Initially, 19,750 adults aged 45 and older were selected. After excluding 2,524 for age non-compliance or missing relevant data, 17,226 participants remained who met the inclusion criteria.

### 2.2 Physical activity assessment

The CHARLS database collected the number of days the respondents were physically active for at least 10 min per day in the past week and the duration of daily activity, and physical activity was assessed using the International Physical Activity Questionnaire-Short Form (IPAQ-SF) (18). Metabolic equivalents (METs) for each physical activity were assigned using the IPAQ, a widely used tool for measuring PAL in adults with demonstrated reliability and validity (18). This method estimated the total weekly energy expenditure of the respondents’ physical activities. MET values of 3.3, 4.0, and 8.0 were, respectively, assigned for low, moderate, and high intensity activities. Total weekly energy expenditure was calculated using the formula: MET × activity time per day (min) × active days per week(d) (24). Physical activity levels were categorized into quartiles for analysis as follows: Q1 ≤ 1732.5 MET/week, 1732.5 < Q2 ≤ 4612.5 MET/week, 4612.5 < Q3 ≤ 10,458 MET/week, Q4 > 10,458 MET/week.

### 2.3 Diabetes assessment

Diabetes assessment was based on self-reported physician diagnosis, high fasting blood glucose levels (fasting blood glucose ≥126 mg/dL or glycated hemoglobin (HbA1c) ≥6.5%) (25), or ongoing antidiabetic treatment. Participants were classified as diabetic based on a positive response to the diabetes diagnosis question.

### 2.4 Covariates

This study’s covariates, selected for their potential impact on diabetes incidence, included age, gender, residency (urban or rural), education level (illiteracy, primary, secondary, and high school and above), health behaviors (alcohol and smoking status), daily living ability, and comorbidities such as hypertension, heart disease, and depression. Depression was assessed using the Center for Epidemiologic Studies Depression Scale (CESD), which consists of 10 entries with 4 answers to each question. The respondent scores each symptom according to the frequency of occurrence of each symptom in the past week, with total scores ranging from 0 to 30, with scores <10 defined as no depression and score ≥ 10 defined as depression (26). The Chinese version of the CESD has been used in the Chinese older adults population, with good reliability and validity, and can effectively measure depression level of middle-aged and older adults people (27).

### 2.5 Data analysis

In this study, data were collated and analyzed using R 4.0.3 software. Measurement data were expressed as mean ± standard deviation (mean ± SD), count data as percentages, categorical variables as frequencies and percentages. Group comparisons were made using the χ2 test or Fisher’s exact test. Binary logistic regression was employed to analyze the relationship between different PAL and diabetes incidence in the middle-aged and older adults population. Model 1 was not adjusted for any variables, model 2 was adjusted for demographic characteristics (age, gender, residency, education level) and health behaviors (smoking status and alcohol status), and model 3 was adjusted for demographic characteristics, health behaviors, daily living ability, and comorbidities (hypertension, heart disease, depression). Subgroup analyses of sex, age and residency were also conducted to explore differences across subgroups. Restricted cubic spline (RCS) was used to assess the dose–response relationship between physical activity and diabetes incidence in the total middle-aged and older adults population and within each subgroup. In addition, sensitivity analyses were used to validate the results for robustness. Results were expressed as odds ratios (ORs) and 95% confidence intervals (CIs), and differences were considered statistically significant when the *p* value was less than 0.05.

## 3 Results

### 3.1 Basic information of the participants

Table 1 shows baseline characteristics for 17,226 middle-aged and older adults participants, with an average age of 61.28 ± 9.71 years. Of these, 8,916 (51.76%) were female and 8,310 (48.24%) were male. There were 7,885 (45.77%) aged 45–59 years, 5,896 (34.23%) aged 60–69 years and 3,445 (20%) aged 70 years and above. Among participants, 2,223 (12.90%) were diagnosed with diabetes, accounting for 12.90% of the total number of people, and the diabetes prevalence is increasing with age. The correlation between diabetes incidence and age, sex, residency, education level, smoking status, drinking status, ability of daily living, hypertension, heart disease, depression, and PAL among the middle-aged and older adults were statistically significant (*p* < 0.05).

**Table 1:** Baseline characteristics of participants.

Variables	No-diabetes (*n* = 15,003)	diabetes (*n* = 2,223)	Total (*n* = 17,226)	*χ* ^2^	*p*
Age (years)				90.677	<0.001
45–59	7,076 (47.16%)	809 (36.39%)	7,885 (45.77%)		
60–69	4,997 (33.31%)	899 (40.44%)	5,896 (34.23%)		
70+	2,930 (19.53%)	515 (23.17%)	3,445 (20%)		
Sex				12.714	<0.001
Female	7,687 (51.24%)	1,229 (55.29%)	8,916 (51.76%)		
Male	7,316 (48.76%)	994 (44.71%)	8,310 (48.24%)		
Residency				94.823	<0.001
Urban	5,907 (39.37%)	1,117 (50.25%)	7,024 (40.78%)		
Rural	9,096 (60.63%)	1,106 (49.75%)	10,202 (59.22%)		
Educational level				22.373	<0.001
Illiteracy	5,859 (39.05%)	832 (37.43%)	6,691 (38.84%)		
Primary school	4,452 (29.67%)	610 (27.44%)	5,062 (29.39%)		
Secondary school	2,952 (19.68%)	450 (20.24%)	3,402 (19.75%)		
High school and above	1740 (11.6%)	331 (14.89%)	2071 (12.02%)		
Smoking status				51.495	<0.001
No	10,746 (71.63%)	1754 (78.9%)	12,500 (72.56%)		
Yes	4,257 (28.37%)	469 (21.1%)	4,726 (27.44%)		
Drinking status				30.696	<0.001
No	9,613 (64.07%)	1,558 (70.09%)	11,171 (64.85%)		
Yes	5,390 (35.93%)	665 (29.91%)	6,055 (35.15%)		
Hypertension				676.085	<0.001
No	9,840 (65.59%)	820 (36.89%)	10,660 (61.88%)		
Yes	5,163 (34.41%)	1,403 (63.11%)	6,566 (38.12%)		
Heart disease				367.117	<0.001
No	12,424 (82.81%)	1,458 (65.59%)	13,882 (80.59%)		
Yes	2,579 (17.19%)	765 (34.41%)	3,344 (19.41%)		
Daily living ability				125.621	<0.001
No	12,737 (84.9%)	1,678 (75.48%)	14,415 (83.68%)		
Yes	2,266 (15.1%)	545 (24.52%)	2,811 (16.32%)		
Depression				24.718	<0.001
No	8,900 (59.32%)	1,195 (53.76%)	10,095 (58.6%)		
Yes	6,103 (40.68%)	1,028 (46.24%)	7,131 (41.4%)		
Physical activity level				139.468	<0.001
Q1	3,823 (25.48%)	761 (34.23%)	4,584 (26.61%)		
Q2	3,494 (23.29%)	581 (26.14%)	4,075 (23.66%)		
Q3	3,760 (25.06%)	517 (23.26%)	4,277 (24.83%)		
Q4	3,926 (26.17%)	364 (16.37%)	4,290 (24.9%)		

Q quartiles; Categorical variables were presented as numbers (percentage).

### 3.2 Binary logistic regression analysis of physical activity and diabetes

This study used diabetes status as the dependent variable and physical activity as the independent variable, incorporating additional covariates in the regression model to assess their relationship. Results are presented in Table 2. The results showed that compared to those in the lowest quartile PAL, participants in the second, third, and fourth quartiles had diabetes prevalence reduced by 16% [OR = 0.84, 95% CI (0.74, 0.94), *p* = 0.003], 31% [OR = 0.69, 95% CI (0.61, 0.78), *p* < 0.001], and 53% [OR = 0.47, 95% CI (0.41, 0.53), *p* < 0.001], respectively. After adjusting for sex, age, residency, education level, smoking status, drinking status, daily living ability, hypertension, heart disease, and depression, participants in the third and fourth quartiles PAL saw diabetes incidence reduced by 16% [OR = 0.84, 95% CI (0.74, 0.95), *p* = 0.005] and 33% [OR = 0.67, 95% CI (0.58, 0.77), *p* < 0.001], respectively, compared to the first quartile. No significant incidence reduction was observed in the second quartile.

**Table 2:** Binary logistic regression analysis for the associations between physical activity and diabetes.

Variables		Model 1		Model 2		Model 3	
	*N*	OR (95% CI)	*p*-value	OR (95% CI)	*p*-value	OR (95% CI)	*p*-value
Q1	4,584	REF		REF		REF	
Q2	4,075	0.84 [0.74, 0.94]	0.003	0.83 [0.74, 0.94]	0.003	0.89 [0.79, 1]	0.059
Q3	4,277	0.69 [0.61, 0.78]	<0.001	0.75 [0.66, 0.84]	<0.001	0.84 [0.74, 0.95]	0.005
Q4	4,290	0.47 [0.41, 0.53]	<0.001	0.57 [0.49, 0.65]	<0.001	0.67 [0.58, 0.77]	<0.001
*p*_for trend_			<0.001		<0.001		<0.001

Data are presented as odds ratio (95% confidence interval); SD standard deviation, Q quartiles, Ref reference.

Model 1 did not adjust for confounders.

Model 2 adjusted for age, sex, residency, education level, smoking status, alcohol status.

Model 3 adjusted for age, sex, residency, education level, smoking status, alcohol status, daily living ability, hypertension, heart disease, depression.

**Figure 1:**
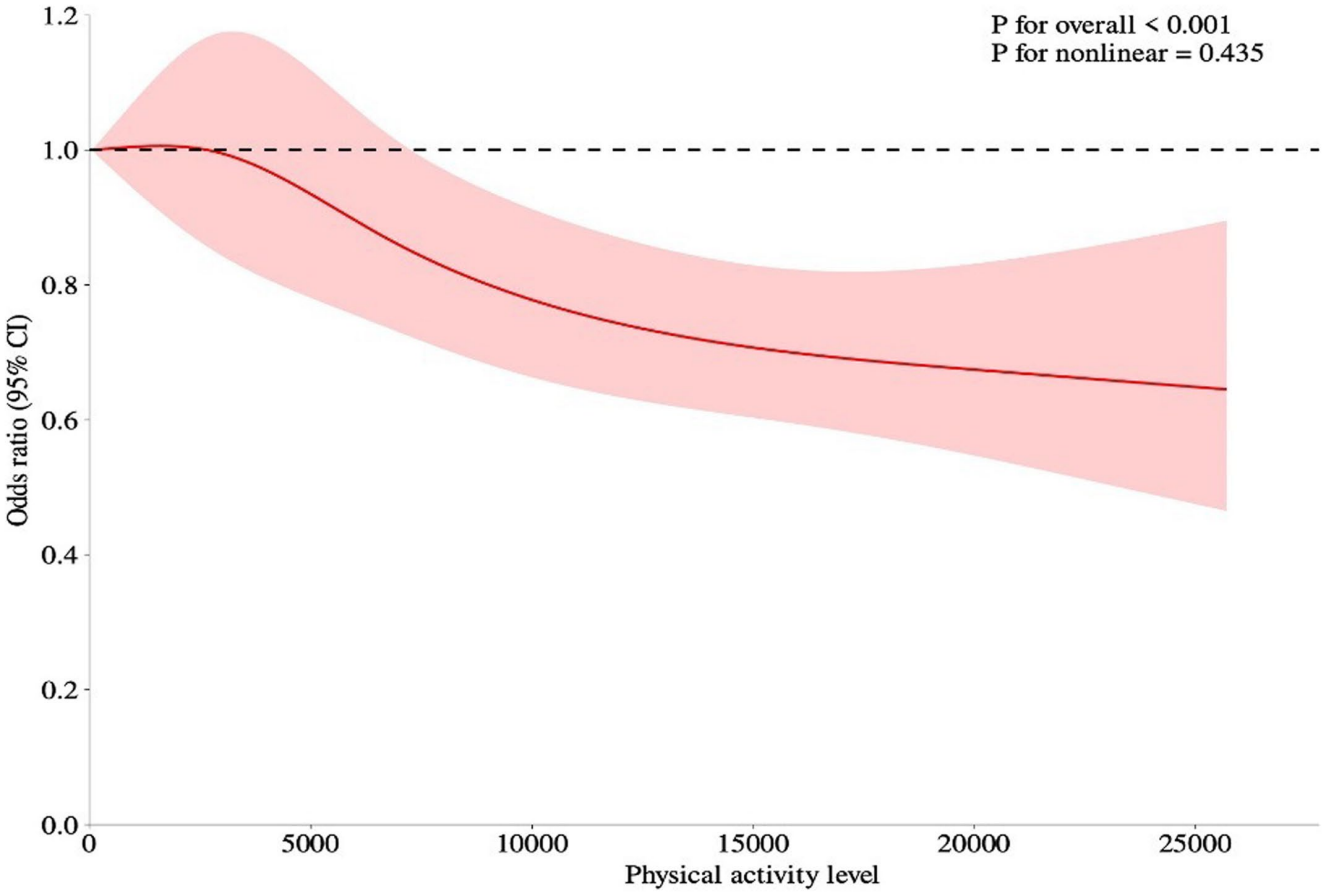
Dose–response relationship between physical activity and the incidence of diabetes in middle-aged and older adults people. The RCS curves were adjusted for age, sex, residency, education level, smoking status, drinking status, daily living ability, hypertension, heart disease, and depression. The solid red lines represented the ORs of diabetes, the red region indicated corresponding 95% CIs. The short dashed black lines indicated the reference value.

In this study, physical activity was introduced as a continuous variable in restricted cubic spline (RCS) curve fitting, and the RCS curve showed a negatively correlated dose–response relationship between physical activity and the diabetes incidence in the middle-aged and older adults population (see Figure 1).

### 3.3 Subgroup analyses

This study conducted subgroup analyses to examine the impact of sex, age, and residency on diabetes incidence among middle-aged and older adults individuals. Age was also categorized into three groups: middle-aged (45–59), early older adults (60–69), and late older adults (70+) to investigate the relationship between physical activity and diabetes incidence in different age intervals. Results are presented in Table 3. Additionally, the RCS analysis was applied to explore the dose–response relationship between physical activity and diabetes incidence within various subgroups. The results are detailed in Supplementary Figures S1–S7.

**Table 3:** Binary logistic analysis for the association between physical activity and diabetes prevalence in various subgroups.

Subgroup	Variables	*N*	OR (95% CI)	*p*-value
Sex				
Female	Q2	2,275	0.88 [0.75, 1.04]	0.133
	Q3	2,379	0.88 [0.75, 1.05]	0.152
	Q4	1912	0.75 [0.62, 0.91]	0.004
	*p*_for trend_			0.008
Male	Q2	1800	0.91 [0.75, 1.09]	0.283
	Q3	1898	0.78 [0.64, 0.95]	0.011
	Q4	2,378	0.59 [0.48, 0.73]	<0.001
	*p*_for trend_			<0.001
Age (years)				
45–59	Q2	1734	0.89 [0.72, 1.1]	0.277
	Q3	2092	0.76 [0.62, 0.94]	0.012
	Q4	2,408	0.61 [0.49, 0.76]	<0.001
	*p*_for trend_			<0.001
60–69	Q2	1,459	0.83 [0.69, 1.01]	0.066
	Q3	1,447	0.83 [0.68, 1.01]	0.063
	Q4	1,447	0.62 [0.49, 0.77]	<0.001
	*p*_for trend_			<0.001
70+	Q2	882	0.95 [0.75, 1.21]	0.68
	Q3	738	0.91 [0.7, 1.19]	0.487
	Q4	435	0.87 [0.6, 1.24]	0.43
	*p*_for trend_			0.354
Residency				
Urban	Q2	2032	0.91 [0.78, 1.07]	0.272
	Q3	1704	0.79 [0.66, 0.95]	0.013
	Q4	1,084	0.59 [0.46, 0.75]	<0.001
	*p*_for trend_			<0.001
Rural	Q2	2043	0.86 [0.72, 1.04]	0.115
	Q3	2,573	0.87 [0.73, 1.04]	0.139
	Q4	3,206	0.72 [0.6, 0.86]	<0.001
	*p*_for trend_			0.001

Data are presented as odds ratio (95% confidence interval); Q quartiles; Q1 was used as the reference; model were adjusted for age, sex, residency, education level, smoking status, drinking status, daily living ability, hypertension, heart disease, and depression.

After adjusting for confounders, compared with the first quartile PAL, the fourth quartile PAL significantly reduced the diabetes incidence among females, individuals aged 60–69 and rural residents by 25% [OR = 0.75, 95% CI (0.62, 0.91), *p* = 0.011], 38% [OR = 0.62, 95% CI (0.49, 0.77), *p* < 0.001] and 28% [OR = 0.72, 95% CI (0.6, 0.86), *p* < 0.001], respectively. Whereas, for males, middle-aged (45–59 years), and urban residents, the third quartile PAL reduced diabetes incidence by 22% [OR = 0.78, 95% CI (0.64, 0.95), *p* = 0.004], 24% [OR = 0.76, 95% CI (0.62, 0.94), *p* = 0.012], 21% [OR = 0.79, 95% CI (0.66, 0.95), *p* = 0.013], respectively. When the fourth quartile PAL was reached, the diabetes incidence was significantly reduced in these populations by 41% [OR = 0.59, 95% CI (0.48, 0.73), *p* < 0.001], 39% [OR = 0.61, 95% CI (0.49, 0.76), *p* < 0.001], and 41% [OR = 0.59, 95% CI (0.46, 0.75), *p* < 0.001], respectively. And there was no statistically significant association between other PAL and diabetes incidence.

### 3.4 Sensitivity analysis

In binary logistic regression analyses, confounding variables may influence the association between physical activity and diabetes incidence, potentially biasing the results. Consequently, this study will assess result robustness by sequentially excluding these confounders. Sensitivity analyses confirmed that the relationship between physical activity and diabetes incidence remained robust (refer to Supplementary Table S1).

## 4 Discussion

In this study, we used a nationally representative cross-sectional study to assess the correlation and dose–response relationship between different PAL and the diabetes incidence in the middle-aged and older adults population in China, and we found that there was a negative dose relationship between physical activity and diabetes incidence, with differences in gender, age, and residency.

The finding that engaging in physical activity reduces the diabetes incidence is consistent with previous research findings (28). Current diabetes prevention guidelines state that individuals can reduce their diabetes incidence through engaging in various physical activities (29). Most previous research has focused on the effects of leisure physical activity on diabetes incidence, with fewer studies on non-leisure physical activity (30). However, in the Chinese middle-aged and older adults population, the proportion of daily participation in leisure physical activity is low (31). Non-leisure physical activities, such as household, commuting, and occupational physical activities, are the main sources of daily energy expenditure. Additionally, it has been suggested that the protective effect of physical activity on diabetes incidence may be related to total energy expenditure (32), not just leisure physical activity. Therefore, this study only examined the relationship between different levels of physical activity and diabetes incidence in middle-aged and older adults without categorizing the types of physical activity.

Several biological mechanisms may explain the beneficial effects of physical activity on diabetes. Initially, physical activity improves energy balance and mitigates obesity (33), which is a major risk factor for diabetes (34, 35). Secondly, muscle contraction improves glucose homeostasis by increasing skeletal muscle glucose uptake by translocating the GLUT4 glucose transporter to skeletal muscle cell membranes and increasing glycogen synthase activity (36, 37). Nextly, physical activity normalizes insulin resistance in liver and skeletal muscles (38). Finally, research has shown that inflammation contributes to the onset of diabetes (39), and physical activity raises interleukin-6(IL-6) levels, which combat inflammation, thus indirectly regulating glycemic irregularities (40, 41). Additionally, Cardiovascular disease is recognized as a risk factor for diabetes (42). Physical activity can increase cardiac efficiency and improve vascular health, thereby reducing the incidence of cardiovascular disease in middle-aged and older adults individuals (43), which is also one of the mechanisms. High PAL have been found to modulate diabetes incidence better than low levels (44–48), aligning with the negative correlation noted in our study between PAL and diabetes incidence. Sustained high PAL can induce adaptations in middle-aged and older adults, such as weight loss, increased GLUT4 and interleukin-6 expression, and reduction in cardiovascular diseases, all contributing to reduced diabetes incidence. However, some studies contrast with our findings, with finding indicating that moderate PAL activity may reduce diabetes incidence more effectively than other levels (20, 49). The reason for this difference may be related to ethnicity, and study found that genetic polymorphisms may be one of the reasons why physical activity has different effects on diabetes incidence in different populations (50). A multiracial cohort study validated this conjecture that differences in gene may have an impact on the relationship between physical activity and diabetes (46). Furthermore, a study provides a possible explanation for this differentiation (51). The interaction between genes and the environment may cause varying rates of fat oxidation during exercise among different ethnic groups, which in turn leads to the need for different PAL in different ethnic groups to modulate diabetes incidence.

Further subgroup analyses revealed gender, age, and residency differences in the effect of physical activity on diabetes among Chinese middle-aged and older adults. Female middle-aged and older adults may require higher PAL than their male counterparts to significantly reduce diabetes incidence, potentially due to differences in gender-induced biological and psychosocial factors (52). At the age level, we found that the beneficial impact of physical activity on diabetes incidence decreases with age, aligning with previous research findings (53). The reason for this phenomenon may be that the increased incidence of diabetes due to aging diminishes the protective effect of physical activity on diabetes (54, 55). At the level of residency, compared to the first quartile PAL, urban middle-aged and older adults experienced significant diabetes incidence reduction at the third and fourth quartile of physical activity, while rural counterparts saw comparable benefits only upon reaching the fourth quartile. Moreover, the same PAL is more effective in reducing diabetes incidence among urban middle-aged and older adults compared to their rural counterparts, a disparity attributed to various factors. A study in India found that (56) urban areas possess superior healthcare systems compared to rural areas, allowing urban older adults to better manage diabetes under the same circumstances. Given that both China and India are developing countries, the disparities between urban and rural areas in China may mirror those in India. It has also been found that differences in self-health management awareness among residents may contribute to the urban–rural disparities (57). Urban middle-aged and older adults individuals may focus on diabetes prevention and treatment earlier than those in rural areas and may take other protective measures to reduce the diabetes incidence when performing the same physical activities. Moreover, there will still be numerous factors contributing to the urban–rural differences, and more further studies are needed to explore the mechanisms in the future.

Several notable strengths exist in this study. Firstly, the data were derived from a representative sample of middle-aged and older adults individuals across China, with a large sample size and a wide range of data collection, encompassing 150 counties and 450 communities in 28 provinces. This broad data collection allows for a comprehensive analysis of the relationship between physical activity and diabetes incidence among this demographic. Secondly, the use of a standardized international questionnaire, combined with a large data sample and sensitivity analyses, enhances the study’s reliability. Thirdly, the study investigates the relationship between various PAL and diabetes incidence, while also analyzing differences across gender, age, and residency. This enriches the research field and provides a valuable reference for future studies.

This study also has several limitations. Firstly, the data were sourced from 17,226 respondents in 2018 without longitudinal data over many years, thus the cross-sectional design could not establish causality between physical activity and diabetes incidence. Secondly, this study focused on a Chinese middle-aged and older adults population, thus its findings are specific to China and may not be generalizable to other countries. Thirdly, this study used self-report questionnaires, and the reporting of physical activity scores and other covariates relied on self-reporting provided by subjects or their informants and is therefore subject to some bias. Fourthly, despite adjustments for several important influencing factors, confounding factors not accounted for may have impacted the study’s results.

## 5 Conclusion

Higher PAL was associated with lower diabetes incidence in specific Chinese middle-aged and older adults population. It is feasible to use physical activity to predict diabetes incidence in this demographic, and high PAL may be an effective means of preventing and controlling diabetes. This study provides the basis and clues for early clinical prediction and reduction of diabetes incidence in middle-aged and older adults people. Future research should intensify efforts to investigate the causal links between physical activity and diabetes incidence, thereby providing a theoretical foundation for diabetes prevention and treatment in the group.

## Data availability statement

The datasets presented in this study can be found in online repositories. The names of the repository/repositories and accession number(s) can be found in the article/Supplementary material.

## Ethics statement

The studies involving humans were approved by Peking University’s Biomedical Ethics Review Committee. The studies were conducted in accordance with the local legislation and institutional requirements. Written informed consent for participation in this study was provided by the participants’ legal guardians/next of kin. Written informed consent was obtained from the individual(s) for the publication of any potentially identifiable images or data included in this article.

## Author contributions

YZ: Conceptualization, Data curation, Investigation, Methodology, Software, Supervision, Writing – original draft, Writing – review & editing. FM: Data curation, Formal analysis, Methodology, Supervision, Writing – original draft, Writing – review & editing. XF: Conceptualization, Data curation, Writing – review & editing. KW: Data curation, Supervision, Writing – review & editing. YW: Conceptualization, Investigation, Writing – review & editing. XW: Conceptualization, Investigation, Software, Writing – original draft, Writing – review & editing.
