# Supplementary material for: Association between physical activity level and diabetes incidence among Chinese middle-aged and older adults: a cross-sectional study from the China health and retirement longitudinal study
Source: Front Public Health. 2024 Aug 9;12:1430229. doi: 10.3389/fpubh.2024.1430229 (PMC11341424; doi:10.3389/fpubh.2024.1430229)
Supplement: Supplementary file 1 [file Data_Sheet_1.PDF]

Supplementary Material

1 Supplementary Figures

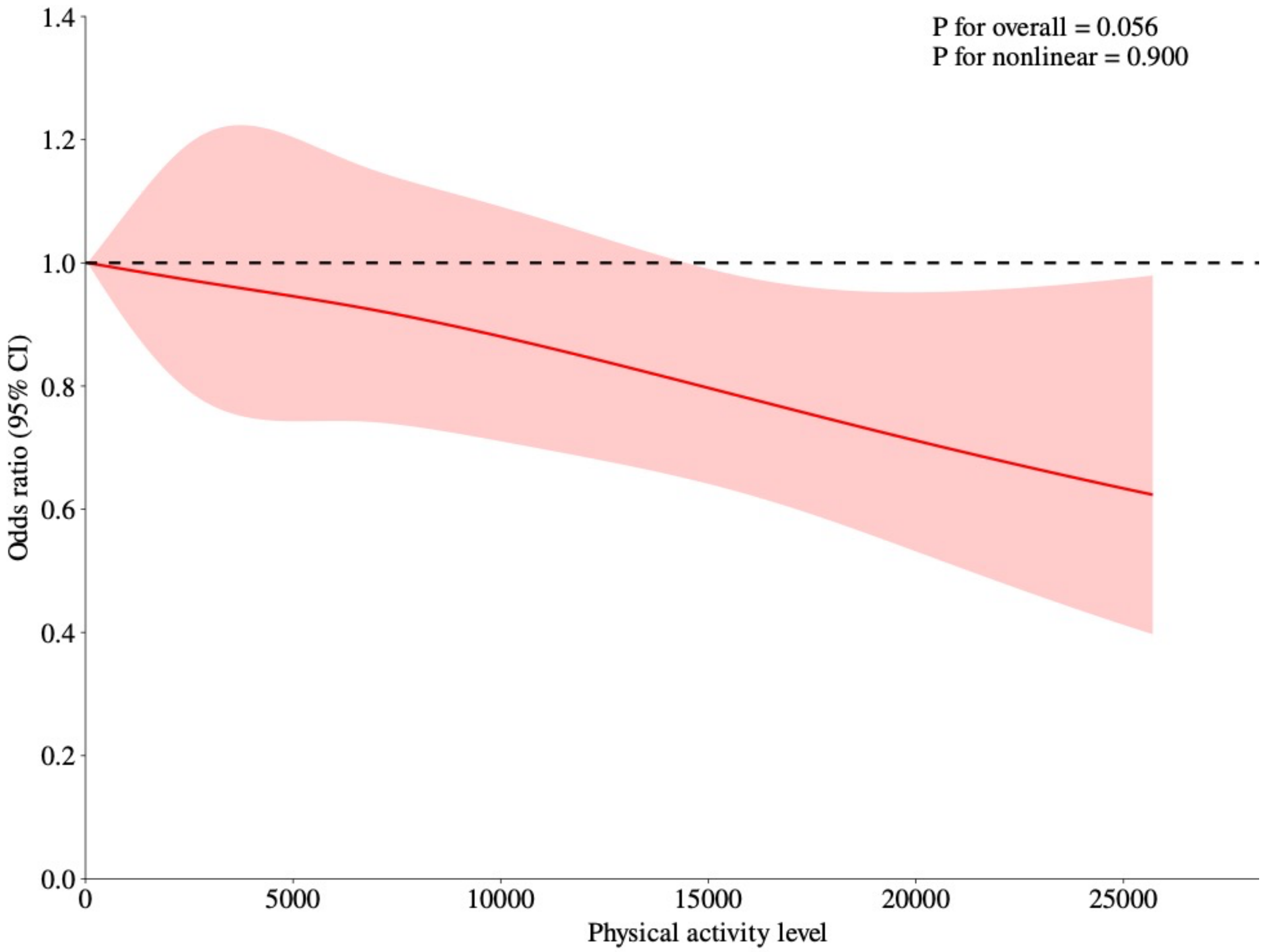

Supplementary Figures 1. Dose-response relationship between physical activity and the incidence of diabetes within woman subgroups.

The RCS curves were adjusted for age, sex, residency, education level, smoking status, drinking status, daily living ability, hypertension, heart disease, and depression.

The solid red lines represented the ORs of diabetes, the red region indicated corresponding 95 % CIs. The short dashed black lines indicated the reference value.

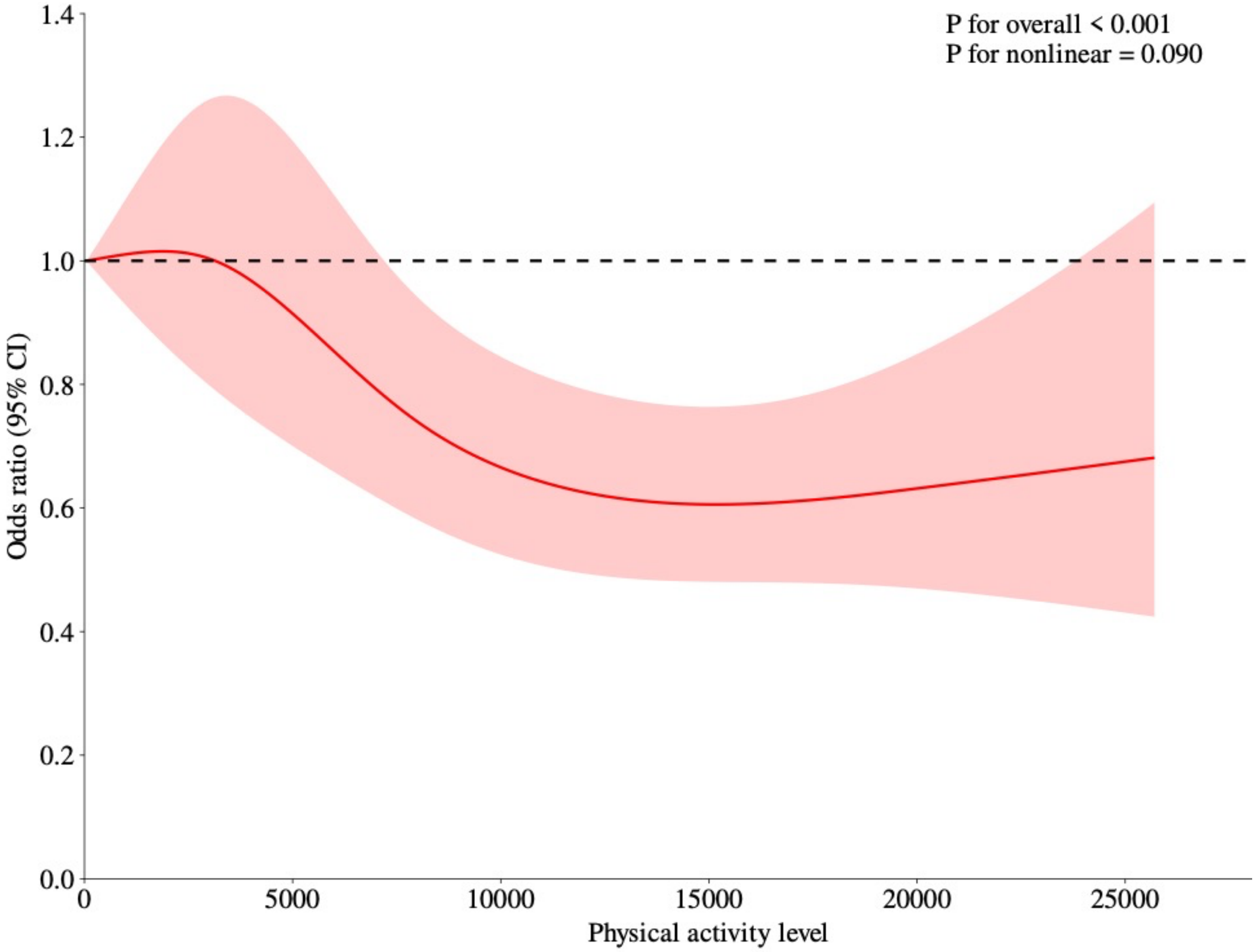

Supplementary Figures 2. Dose-response relationship between physical activity and the incidence of diabetes within man subgroups.

The RCS curves were adjusted for age, sex, residency, education level, smoking status, drinking status, daily living ability, hypertension, heart disease, and depression.

The solid red lines represented the ORs of diabetes, the red region indicated corresponding 95 % CIs. The short dashed black lines indicated the reference value.

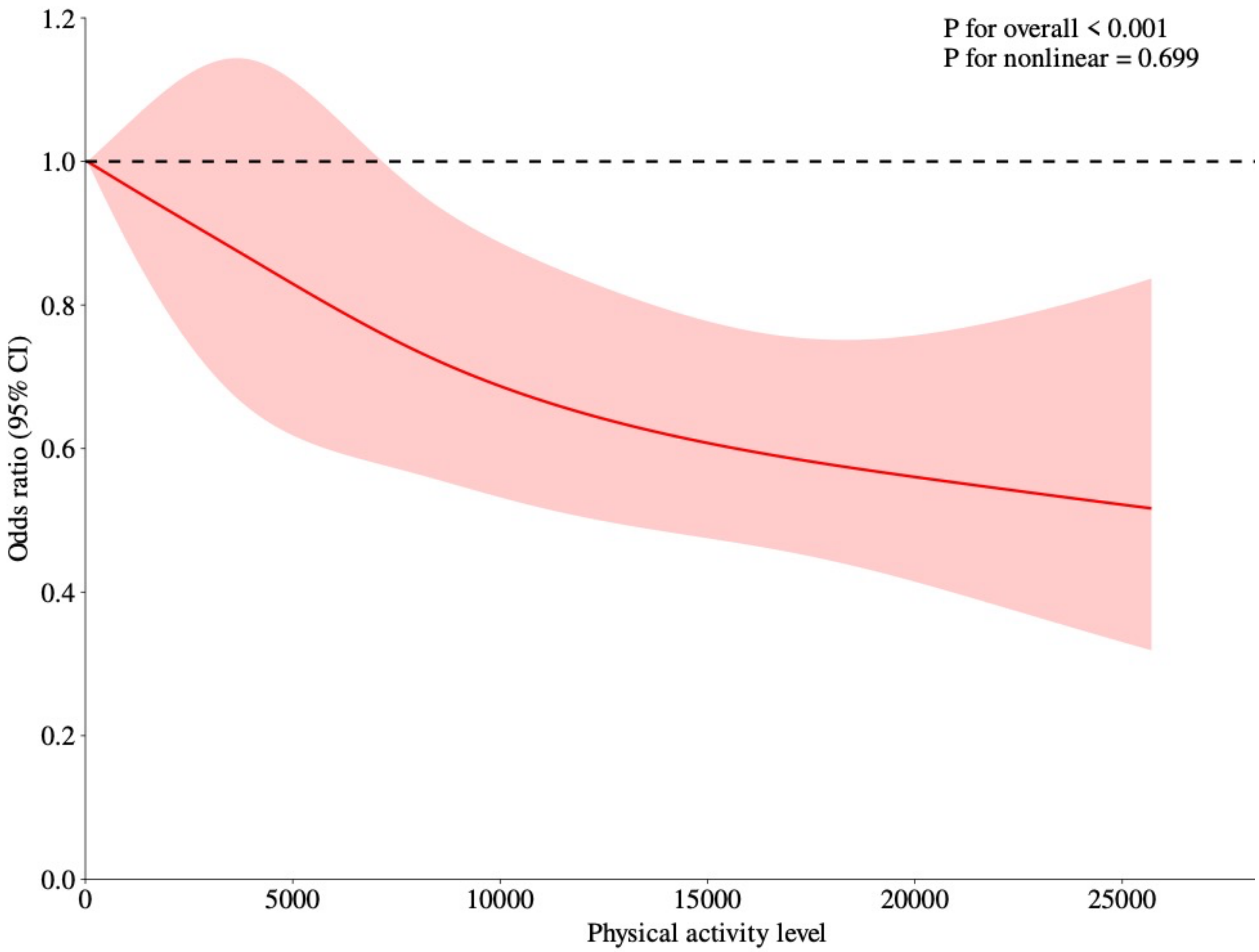

Supplementary Figures 3. Dose-response relationship between physical activity and the incidence of diabetes within middle aged (45-59) subgroups.
The RCS curves were adjusted for age, sex, residency, education level, smoking status, drinking status, daily living ability, hypertension, heart disease, and depression.
The solid red lines represented the ORs of diabetes, the red region indicated corresponding 95 % CIs. The short dashed black lines indicated the reference value.

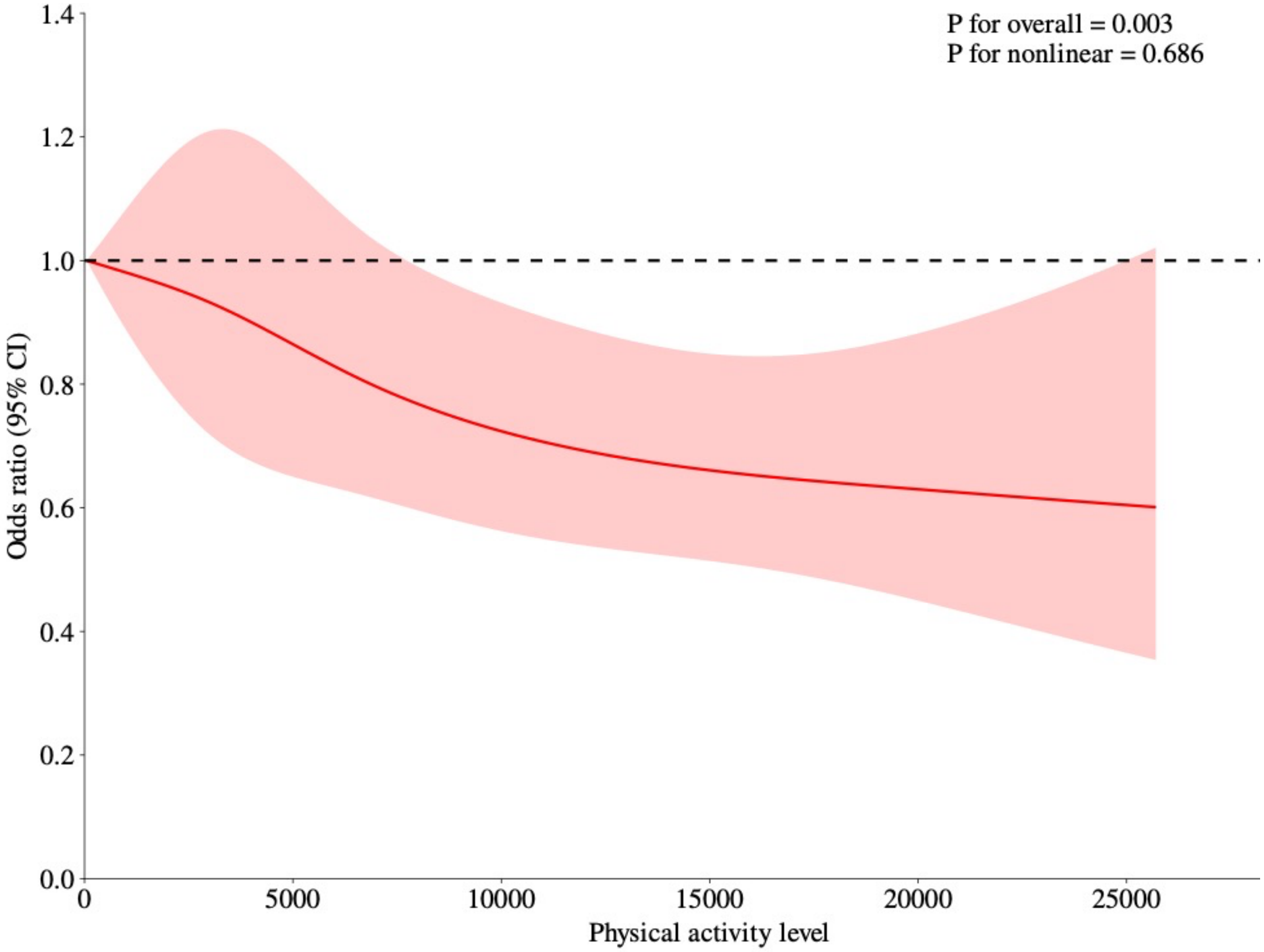

Supplementary Figures 4. Dose-response relationship between physical activity and the incidence of diabetes within older adults aged 60-69 subgroups.

The RCS curves were adjusted for age, sex, residency, education level, smoking status, drinking status, daily living ability, hypertension, heart disease, and depression.

The solid red lines represented the ORs of diabetes, the red region indicated corresponding 95 % CIs. The short dashed black lines indicated the reference value.

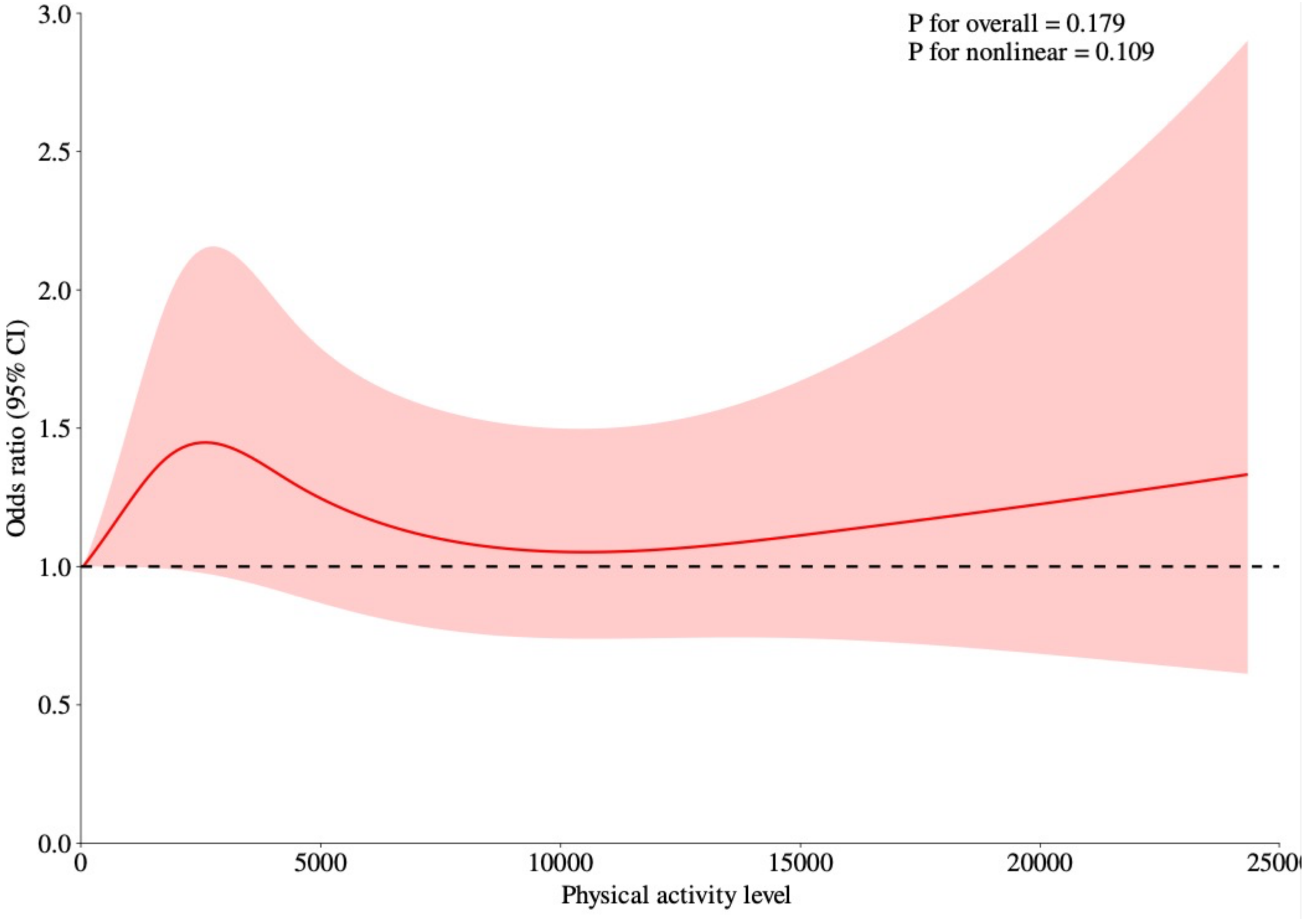

Supplementary Figures 5. Dose-response relationship between physical activity and the incidence of diabetes within older adults aged 70 and above subgroups.

The RCS curves were adjusted for age, sex, residency, education level, smoking status, drinking status, daily living ability, hypertension, heart disease, and depression.

The solid red lines represented the ORs of diabetes, the red region indicated corresponding 95 % CIs. The short dashed black lines indicated the reference value.

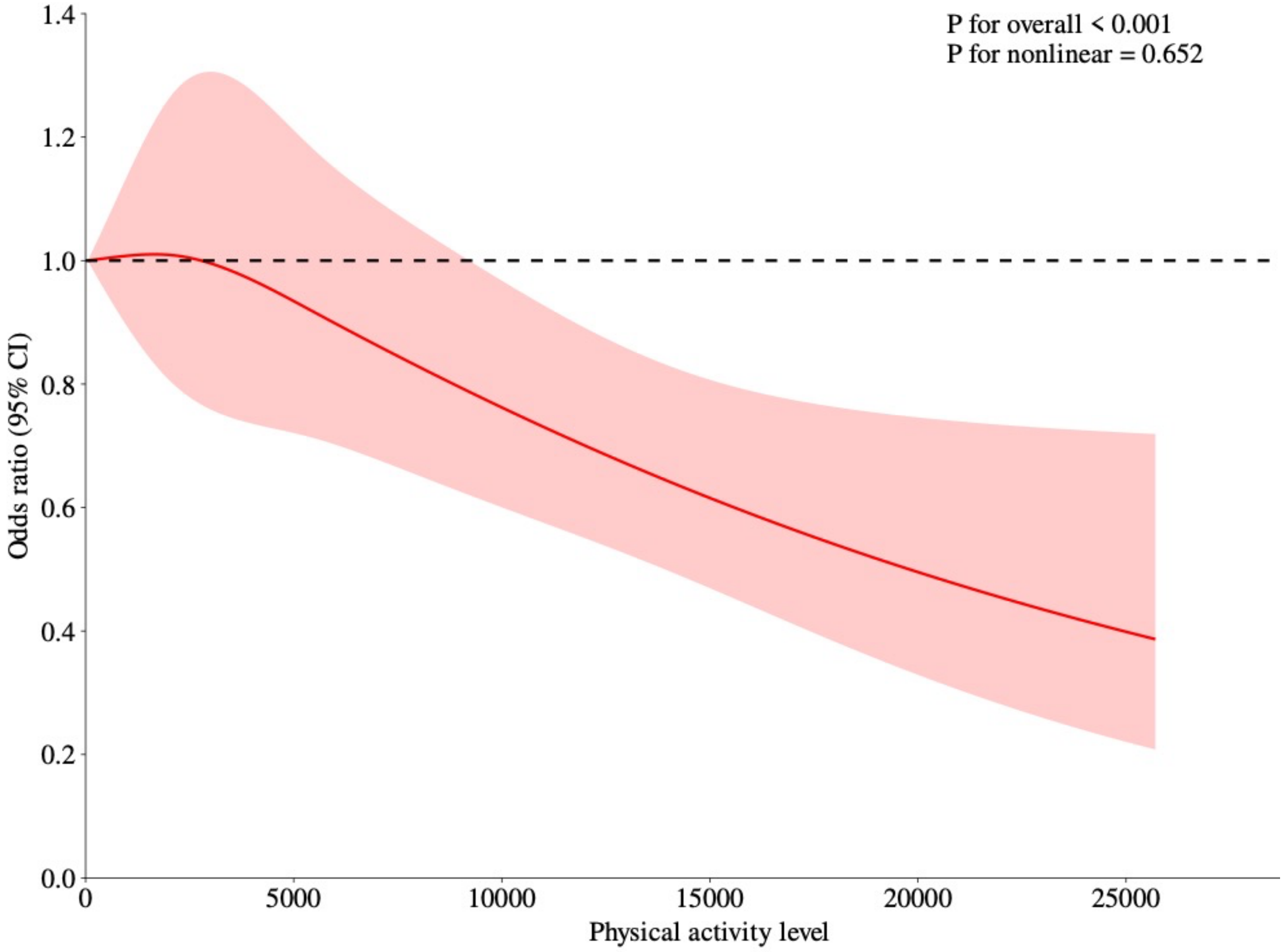

Supplementary Figures 6. Dose-response relationship between physical activity and the incidence of diabetes within urban subgroups.
The RCS curves were adjusted for age, sex, residency, education level, smoking status, drinking status, daily living ability, hypertension, heart disease, and depression.
The solid red lines represented the ORs of diabetes, the red region indicated corresponding 95 % CIs. The short dashed black lines indicated the reference value.

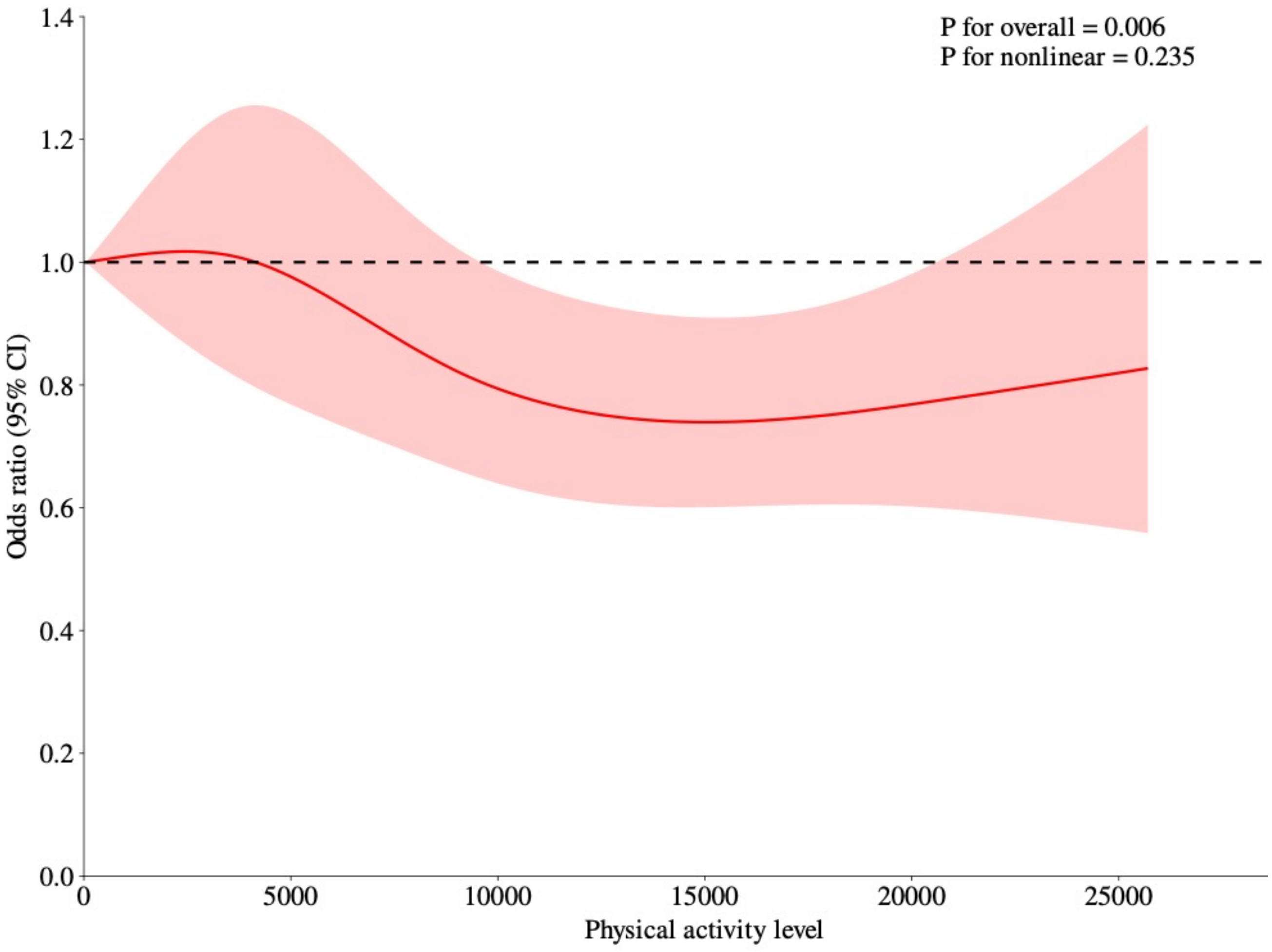

Supplementary Figures 7. Dose-response relationship between physical activity and the incidence of diabetes within rural subgroups.

The RCS curves were adjusted for age, sex, residency, education level, smoking status, drinking status, daily living ability, hypertension, heart disease, and depression.

The solid red lines represented the ORs of diabetes, the red region indicated corresponding 95 % CIs. The short dashed black lines indicated the reference value.

2 Supplementary Tables

Supplementary Table 1. Sensitivity analyses of the association between PAL and diabetes incidence, after excluding Confounding factor.

PAL		Model 1		Model 2		Model 3		Model 4		Model 5	
	N	OR (95%CI)	P-value	OR (95%CI)	P-value	OR (95%CI)	P-value	OR (95%CI)	P-value	OR (95%CI)	P-value
Q1	4584	REF		REF		REF		REF		REF	
Q2	4075	0.9[0.8,1.02]	0.086	0.89[0.79,1]	0.055	0.89[0.79,1.01]	0.062	0.89[0.79,1.01]	0.063	0.9[0.79,1.01]	0.075
Q3	4277	0.84[0.74,0.95]	0.007	0.83[0.74,0.95]	0.005	0.81[0.72,0.92]	0.001	0.83[0.73,0.94]	0.003	0.84[0.74,0.95]	0.006
Q4	4290	0.67[0.59,0.78]	<0.001	0.67[0.58,0.77]	<0.001	0.62[0.54,0.72]	<0.001	0.65[0.57,0.75]	<0.001	0.67[0.58,0.77]	<0.001
P for trend			<0.001		<0.001		<0.001		<0.001		<0.001

PAL		Model 6		Model 7		Model 8		Model 9		Model 10	
	N	OR (95%CI)	P-value	OR (95%CI)	P-value	OR (95%CI)	P-value	OR (95%CI)	P-value	OR (95%CI)	P-value
Q1	4584	REF		REF		REF		REF		REF	
Q2	4075	0.89[0.78,1]	0.052	0.88[0.78,0.99]	0.031	0.88[0.78,1]	0.044	0.87[0.77,0.99]	0.028	0.89[0.78,1]	0.053
Q3	4277	0.83[0.73,0.94]	0.004	0.8[0.71,0.91]	<0.001	0.82[0.72,0.93]	0.002	0.82[0.72,0.93]	0.002	0.83[0.73,0.95]	0.005
Q4	4290	0.66[0.57,0.76]	<0.001	0.62[0.54,0.72]	<0.001	0.65[0.56,0.75]	<0.001	0.65[0.56,0.75]	<0.001	0.67[0.58,0.77]	<0.001
P for trend			<0.001		<0.001		<0.001				<0.001

Model 1 excluding age.
Model 2 excluding gender.
Model 3 excluding residency.
Model 4 excluding education level.
Model 5 excluding Smoking status.
Model 6 excluding Drinking status.
Model 7 excluding Hypertension.

Model 8 excluding heat disease.
Model 9 excluding daily living ability.
Model 10 excluding depression.
